# Cytoplasmic organization promotes protein diffusion in *Xenopus* extracts

**DOI:** 10.1038/s41467-022-33339-0

**Published:** 2022-09-23

**Authors:** William Y. C. Huang, Xianrui Cheng, James E. Ferrell

**Affiliations:** 1 Department of Chemical and Systems Biology, Stanford University School of Medicine, Stanford, CA 94305 USA; 2 Department of Biochemistry, Stanford University School of Medicine, Stanford, CA 94305 USA; 3 Present Address: Department of Biological Sciences, University of Southern California, Los Angeles, CA 90089 USA

**Keywords:** Molecular biophysics, Cell biology, Proteins

## Abstract

The cytoplasm is highly organized. However, the extent to which this organization influences the dynamics of cytoplasmic proteins is not well understood. Here, we use *Xenopus laevis* egg extracts as a model system to study diffusion dynamics in organized versus disorganized cytoplasm. Such extracts are initially homogenized and disorganized, and self-organize into cell-like units over the course of tens of minutes. Using fluorescence correlation spectroscopy, we observe that as the cytoplasm organizes, protein diffusion speeds up by about a factor of two over a length scale of a few hundred nanometers, eventually approaching the diffusion time measured in organelle-depleted cytosol. Even though the ordered cytoplasm contained organelles and cytoskeletal elements that might interfere with diffusion, the convergence of protein diffusion in the cytoplasm toward that in organelle-depleted cytosol suggests that subcellular organization maximizes protein diffusivity. The effect of organization on diffusion varies with molecular size, with the effects being largest for protein-sized molecules, and with the time scale of the measurement. These results show that cytoplasmic organization promotes the efficient diffusion of protein molecules in a densely packed environment.

## Abstract (web summary)

Cytoplasmic organization is a hallmark of living cells. Here, the authors make use of self-organizing cell extracts to examine how the emergence of large-scale organizations influences the microscopic diffusion of protein molecules in the cytoplasm.

## Introduction

The cytoplasm is a crowded environment filled with macromolecules and organelles^1–3^. Its density is high enough to make intracellular motion exhibit glassy dynamics under some conditions^4,5^. At a first glance, one might expect biochemical reactions in such an environment to be slow and inefficient. Nevertheless, protein motion in living cells is fast enough to allow reactions to occur on physiological timescales.

The cytoplasm maintains a spatial organization specific to cell identity and the cell cycle phase. It seems possible that the organization of the cytoplasm affects protein diffusion, which in turn could affect the rate of all processes for which diffusion is at least partially rate limiting^6^. For example, the time it takes for a transcription factor to find the enhancer(s) to which it binds partly depends upon how fast the transcription factor can diffuse^7^. Diffusion partially determine how fast trigger waves of protein activity can propagate^8,9^, and how quickly and on what distance scales patterns can be produced by reaction-diffusion mechanisms^10^. Diffusion determines the dynamics of phase separation and the critical concentrations of species required to form a stable condensate^11,12^. Classic work has shown that actin polymerization is diffusion-limited^13^, and recent work indicates that in yeast, cytoplasmic viscosity and protein diffusion rates are homeostatically maintained^14^. Thus, although almost all metabolic enzymes appear to be reaction-limited rather than diffusion limited^15^, it seems likely that protein diffusion rates could broadly influence many cellular processes.

*Xenopus laevis* egg extracts are essentially undiluted cell-free cytoplasm that retains many of the biological functions of cells^16–19^; for example, extracts can carry out cell cycles^18,20–22^ and can undergo well-organized waves of apoptosis^23^. Extracts can also be homogenized more thoroughly than intact living cells can. Moreover, recent work has shown that even well-mixed interphase extracts can spontaneously generate cell-like spatial organization from a disorganized state, forming polygonal units rich in microtubules and organelles separated by organelle-poor border regions^24–26^. Here we made use of these extracts to determine how cytoplasmic organization affects protein diffusion.

## Results

**Fig. 1:**
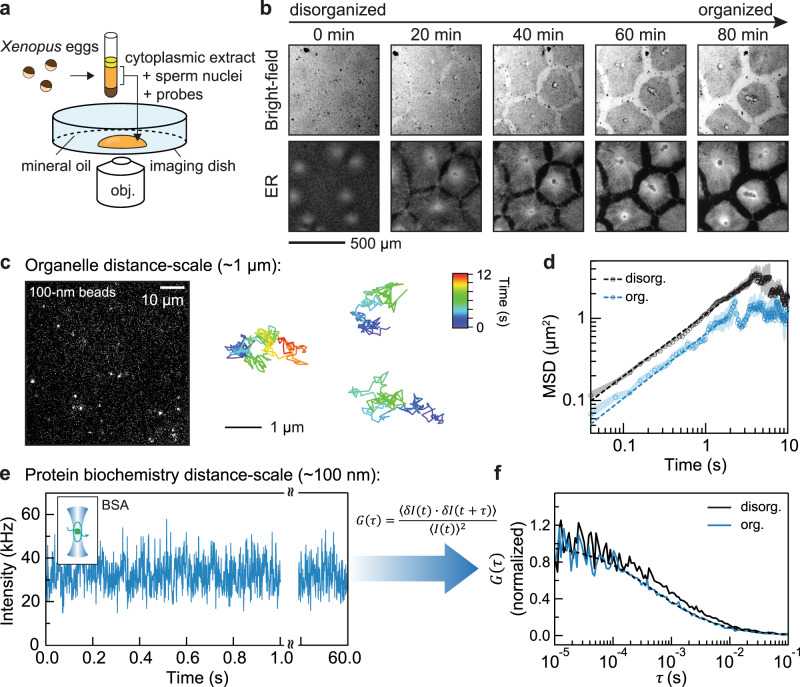
Resolving molecular diffusion in cytoplasmic extracts. **a** Schematic of the experimental setup used in this study: *Xenopus laevis* eggs were fractionated to obtain the undiluted cytoplasmic extract, which was then deposited on the surface of an imaging dish and covered with mineral oil. **b** Time-lapse images of the self-organizing cytoplasm, visualized with 1 µM ER-Tracker Red dye. **c** Image (left) of dilute 100-nm (diameter) microspheres in extracts. Trajectories (right) are examples from single-particle tracking in the organized cytoplasm (interior regions in Fig. 2a). The relative positions between the three tracked particles were adjusted for display (they were tens of microns apart in the extracts). **d** Ensemble mean squared displacement (MSD) analysis derived from the single-particle tracking. Shaded area, standard error of the mean (SEM). Dashed lines are fitting to ${MSD}\left(t\right)=\Gamma {t}^{\alpha }$, where Γ is the generalized diffusion coefficient^34^. The fitted {Γ, *α*} for disorganized and organized cytoplasm are {1.12, 0.75}, and {0.65, 0.78}, respectively. The localization error of these SPT experiments, quantified with immobilized microspheres, was below 0.01 µm^2^ (Supplementary Fig. 1a). **e**, **f** Representative FCS data of BSA-Alexa Flour 488 in cytoplasmic extracts. The full intensity trajectory (60 s) is shown in Supplementary Fig. 2c. The inset depicts a molecule diffusing through a confocal spot. *G*(*τ*) is the autocorrelation function, where *τ* is the lag time; $\delta I\left(t\right)=I\left(t\right)-\left\langle I\left(t\right)\right\rangle$, where *I*(*t*) is the fluorescence intensity and the brackets denote time averages. The autocorrelation curve was fitted by an anomalous diffusion model (dashed line). In this example, the number of particles was 42, and the average intensity (shown in **e**) was 32 kHz, corresponding to a molecular brightness of 0.76 kHz/molecule. Source data are provided as a Source Data file.

We prepared an interphase-arrested cytoplasmic extract supplemented with demembranated *Xenopus* sperm nuclei and an endoplasmic reticulum (ER) dye, as described^27^, deposited the extract in an imaging dish covered with bioinert mineral oil^24,28,29^ (Fig. 1a), and monitored its organization by bright-field and fluorescence microscopy (Fig. 1b). At early time points (<20 min), the homogenized extract showed no apparent long-range organization; organelles, including the ER, were homogeneously dispersed throughout the extract. Over the course of 20–60 min, the extract self-organized into a sheet of cell-like units (Fig. 1b). The interiors of these units were enriched in ER and other organelles; the nuclei were positioned close to the center of the interior; and separating the individual units were borders depleted of organelles. Similar results have been previously described^24^.

### Particles diffuse slower over micron distances after self-organization

We first characterized diffusion on the distance-scale of typical organelles (~1 µm) with single-particle tracking (SPT) (Fig. 1c). Since organelles became enriched in the interior of the cell-like units during self-organization, one might expect that diffusion over this scale would slow down due to the increased concentration of obstacles. SPT of 100-nm (diameter) microspheres indeed showed slower diffusion in organized cytoplasm (Fig. 1d, Supplementary Fig. 1a), suggesting that organization of organelles and/or cytoskeleton elements impeded diffusion of these relatively large microspheres across a distance-scale of microns.

### Protein diffusion speeds up during self-organization

We then asked how cytoplasmic organization affects the diffusion of protein molecules on a biochemical length-scale (a few hundred nm). To put this distance scale into perspective, a protein *X* that functions through its interactions with a moderately-scarce, uniformly distributed protein *Y* (with a concentration of, say, ~200 nM, or about 1/25000 of the total protein concentration in cytoplasm^30^) would need to traverse a distance of about 200 nm to find its target. Thus, this nanometer length scale is relevant to many dynamical protein-protein interactions. It is not obvious whether proteins diffusing on this length scale would behave like microspheres diffusing over microns, because most proteins are only a few nanometers in size and may better percolate through organelle and cytoskeleton structures.

**Fig. 2:**
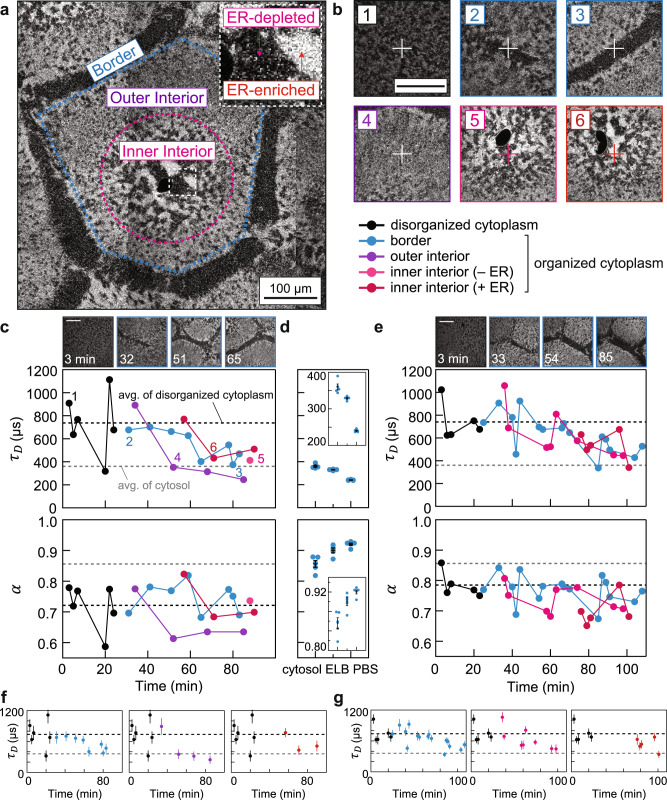
Dynamical transition of short-range diffusion in cytoplasmic extracts. **a**, Spatial regions of self-organization (color-coded), classified by the ER morphology. The dark sphere near the center of the inner interior is the nuclei. The image was stitched from multiple confocal images. **b** Representative confocal images of FCS foci, which are marked by the crosshairs. The annotated numbers refer to snapshots taken prior to FCS measurements in **c**. **c** Tracing protein diffusion during self-organization by FCS. The plots show the characteristic diffusion times *τ*_*D*_ and the diffusion mode *α* obtained from fitting the FCS curves. Black and gray dashed lines correspond to averages from disordered cytoplasmic extracts (in this example, 740 µs, *D*_*eff*_ = 11.9 µm^2^/s) and cytosolic extracts (360 µs, *D*_*eff*_ = 24.7 µm^2^/s), respectively. **d** Benchmark measurements from cytosolic extracts and buffers. ELB, egg lysis buffer; PBS, Phosphate-buffered saline. Insets show zoom-in of the data. Each condition was measured five times; all data presented by error bars are mean ± SEM. *n* = 5 measurements of different positions in a sample. **e**, Biological repeat of **c**, prepared from a separate batch of eggs. **f**, **g** are isolated traces from **c** and **e**. Error bars, estimated errors (standard deviation) from curve fitting (each fitting was performed over *n* = 107 data points). Scale bars, 100 µm. Source data are provided as a Source Data file.

We used fluorescence correlation spectroscopy (FCS) to quantify protein dynamics in the self-organizing extract over this shorter distance scale and faster timescale (Fig. 1e, f). FCS detects individual fluorescent molecules diffusing in and out of a diffraction-limited confocal spot (~400 nm); the fluorescence fluctuations enable the determination of molecular diffusion on a microsecond-to-millisecond timescale by computing the autocorrelation function^31^. We initially chose bovine serum albumin (BSA) labeled with Alexa Fluor 488 as the probe. In a typical experiment, we placed an organizing extract on the microscope stage and first acquired confocal images to characterize the local spatial organization. We then immediately obtained FCS data to measure the local dynamics at specific locations within that field (Fig. 2a, b). As self-organized structures emerged, we collected FCS data at locations within the border region, which is depleted of ER; the outer interior, which has a contiguous ER morphology; and the inner interior, which consists of both ER-depleted and ER-enriched areas (Fig. 2a).

We found that an anomalous diffusion model was required to describe the FCS curves measured in all cytoplasmic extracts, and we obtained the characteristic diffusion time *τ*_*D*_ and the parameter *α* (Fig. 1f, Supplementary Fig. 2, 3)^32–34^. The value of *α* characterizes the diffusion mode as defined in the mean squared displacement (MSD) equation, *MSD*∝*t*^*α*^. For Brownian motion, *α* = 1, which is typically the case for BSA or dye molecules diffusing in buffers. As a control, we verified that the diffusion measurements of microspheres in buffers yielded near-Brownian motion (*α* = 0.97), indicating that any imperfection in the optics did not measurably distort the type of motion (Supplementary Fig. 2). Molecular diffusion in complex materials like cytoplasm is often subdiffusive, characterized by a sublinear dependence of MSD on time, with *α* *<* 1. This subdiffusivity has been attributed to molecular crowding and/or long-scale confinement^1,32–35^.

Cytoplasmic self-organization was accompanied by striking changes in protein diffusivity. Two representative experiments are shown in Fig. 2c, e, with a third example shown in Supplementary Data Fig. 4. In the initially disorganized cytoplasm, the BSA dynamics were subdiffusive, and the average value of *τ*_*D*_ was 677 ± 29 µs (mean ± SEM, *n* = 6 biologically independent samples). This *τ*_*D*_ value corresponds to an effective Brownian diffusion coefficient *D*_*eff*_ of 13.2 µm^2^/s (by using the relation ${D}_{{eff}}={w}^{2}/(4{\tau }_{D}),$ where *w* is the confocal radius). As the border regions emerged and became depleted of organelles, the diffusion time within these regions decreased by almost a factor of 2 (Fig. 2c, e–g). Unexpectedly, the diffusion time also decreased, and to a similar extent, in the interior regions. Overall, the average *τ*_*D*_ for the organized cytoplasmic extracts fell to 404 ± 35 µs (mean ± SEM, *n* = 6; *D*_*eff*_ = 22.1 µm^2^/s), and there were no significant differences between the values in the border regions vs. interior regions.

We next compared the diffusion times in organized cytoplasm to those in organelle-depleted cytosolic extracts (Fig. 2d, Supplementary Fig. 3; gray lines in Fig. 2c, e–g). Cytosolic extracts were prepared identically to cytoplasmic extracts, except with an additional centrifugation step that removes membrane organelles^27^. BSA diffusion in cytosolic extracts had a characteristic time of 310 ± 25 µs (mean ± SEM, *n* = 3; *D*_*eff*_ = 28.8 µm^2^/s) with no detectable changes over an hour. This is about a factor of 2 smaller than the diffusion time in disorganized organelle-containing cytoplasm (677 µs), and was close to the diffusion times seen in these extracts once they had become organized (404 µs). This suggests that once the cytoplasm has organized its microtubules and organelles, protein diffusion is nearly as fast as it can be for a solution of this protein composition and concentration. In this sense, protein diffusion appears optimized or maximized.

**Fig. 3:**
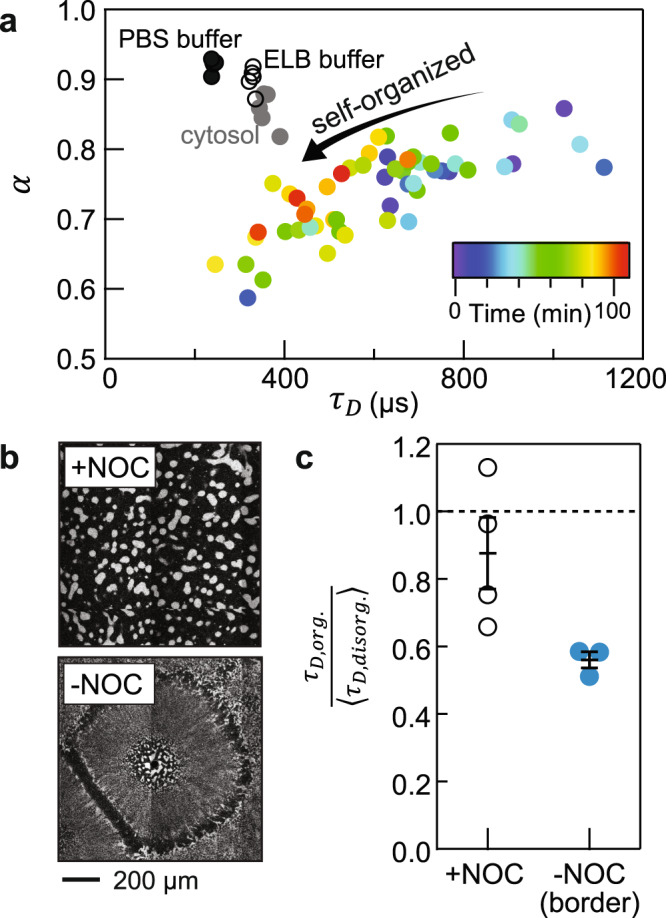
Cytoplasmic organization mediates protein diffusion. **a** FCS data from Fig. 2c–e consolidated in a parameter space of {*α*, *τ*_*D*_}. **b** Nocodazole (NOC, 33 µM) abolishes self-organization of cytoplasm, visualized by ER-Red Tracker. **c** Nocodazole-treated cytoplasmic extracts show little changes in diffusion time (diffusion time ratio close to 1), compared with the sample without nocodazole at similar timepoints (<5 min). The *y*-axis shows *τ*_*D*_ normalized by the average *τ*_*D*_ from early timepoints (<20 min). Error bars, SEM. *n* = 4 and three measurements of different positions in an extract with and without nocodazole, respectively. Source data are provided as a Source Data file.

Diffusion in the cytoplasmic extracts (*α* ≈ 0.75; Fig. 2c, e) was more subdiffusive than that in the cytosolic extracts (*α* ≈ 0.85; Mann–Whitney *U*-test, two tailed *p*-value = 0.015; Fig. 2d, Supplementary Fig. 3), suggesting that microtubules and/or organelles, even when organized, constitute barriers that hinder long-range diffusion. Overall there appeared to be a slight trade-off between *α* and *τ*_*D*_ in cytoplasmic extracts: as the cytoplasm self-organized, diffusion became faster but also more subdiffusive (Fig. 3a). The slightly more subdiffusive nature of the diffusion suggests that organized organelles are less of an obstacle for protein movements over shorter distance scales than over longer distance scales, both in the border and the interior regions. A similar trade-off between the diffusion time and the subdiffusion parameter has been documented in mitotic HeLa cells^36^.

Substantial variation was found in *α* and *τ*_*D*_ for different foci within the same microscopy field. This variation could be due to error in the FCS measurements and/or variability in the heterogeneous extract’s dynamics. To determine how much of this variation was likely to be due to measurement error, we carried out measurements of BSA diffusion in phosphate-buffered saline (PBS), egg lysis buffer (ELB, which includes sucrose, which slows diffusion), and cytosolic extracts. The standard deviation (SD) of the *τ*_*D*_ values were about 4 µs (coefficient of variation CV = SD/mean = 1.6%), 5 µs (1.5%), and 18 µs (5.0%), respectively (Fig. 2d, Supplementary Fig. 3). Additionally, we estimated the upper bound of measurement error in cytoplasmic extracts to be ~50 µs (CV = 15%) based on repeated measurements of the same position in a mature border region (Supplementary Fig. 5). The variation in our experiments (~20–30%) was larger than the measurement error (<15%), suggesting that additional variation between different positions reflects the degree of local organization. Note though that the overall trends in the data—decreases in the *τ*_*D*_ values as self-organization proceeded—were found in all three replicate experiments and in all regions of the self-organizing cytoplasm examined (Fig. 2c, e, Supplementary Fig. 4).

As previously shown, extracts treated with the microtubule poison nocodazole do not undergo self-organization on our experimental timescale (Fig. 3b)^24^. Correspondingly, BSA diffusion showed little change over time in nocodazole-treated extracts (Fig. 3c; statistical tests are shown in Supplementary Fig. 6), consistent with the hypothesis that the cytoplasmic organization is essential for the faster diffusion.

### The diffusion effects are specific to protein-sized molecules

**Fig. 4:**
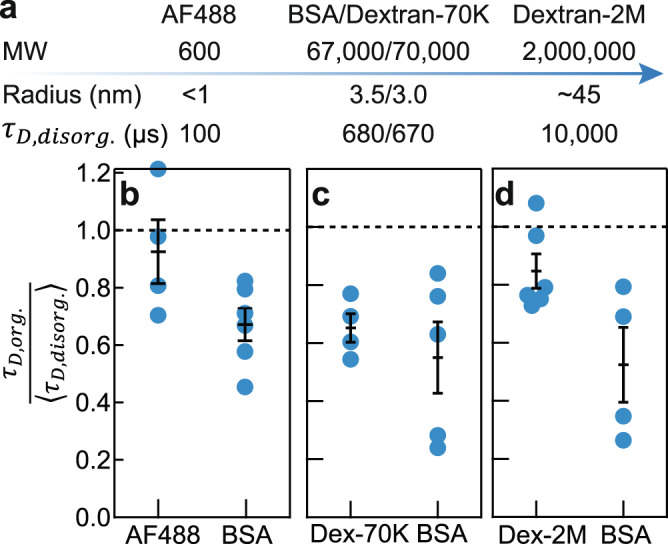
Organized cytoplasm optimizes short-range diffusion of protein-sized molecules. **a** Molecular sizes of probes used in this study. **b**–**d** Normalized diffusion times of Alexa Fluor 488 dye (AF488), dextran-MW70,000 (Dex-70K), and dextran-MW2,000,000 (Dex-2M) in organized cytoplasmic extracts. These experiments focused on the border regions of the organized cytoplasm such that data collected <10 min could be better compared. Each panel shows data from two droplets of extracts prepared from a single batch of eggs; BSA diffusion was taken as a standard for comparison. Error bars, SEM. *n* = 4, 6, 4, 5, 6, and 4 measurements of different positions in an extract for data from left to right, respectively. Source data are provided as a Source Data file.

We then asked if the faster diffusion in organized cytoplasm on the FCS timescale is true for particles of all sizes, or only for those within a specific size range. As benchmarks, we measured Alexa Fluor 488 dye and dextran-MW2,000,000 (referred to as AF488 and dextran-2M, respectively) in the disorganized and organized cytoplasm; the Stokes radii, characterized by FCS in buffers, of AF488, BSA, and dextran-2M are about <1 nm, 3.5 nm, and 45 nm, respectively (Fig. 4a, Supplementary Fig. 7)^37^. These differently sized molecules exhibited distinct dynamics in the cytoplasm in terms of diffusion timescales and degrees of subdiffusivity. In disorganized cytoplasm, the diffusion times of AF488, BSA, and dextran-2M were about 100, 680, and 10,000 µs, respectively (*D*_*eff*_ ~ 90, 15, 1 µm^2^/s, respectively). In terms of the diffusion mode, BSA was the most subdiffusive (*α* ≈ 0.75); dextran-2M was less subdiffusive (*α* ≈ 0.85) and AF488 was nearly Brownian (*α* ≈ 0.95).

Intriguingly, only protein-sized molecules were strongly affected by self-organization. Despite having greatly different timescales (100 µs versus 10,000 µs), the diffusion times of both AF488 and dextran-2M exhibited very little dependence on self-organization at a short distance-scale (Fig. 4b, d; statistical tests are shown in Supplementary Fig. 8). The dextran-2M result contrasts with the SPT data for similar sized 100-nm microspheres, which diffused more rapidly in disorganized extracts than in organized extracts (Fig. 1d). However, the FCS diffusion time of dextran-2M was approximately in line with the SPT data (measured with 100-nm microspheres) when the data were plotted together on the same axes (Supplementary Fig. 1b). Dextran-2M became perhaps slightly less subdiffusive in ordered cytoplasm (*α* **~** 0.85 → 0.90); two-tailed (*p*-value = 0.18) and AF488 remained diffusive throughout the experiments (*α* **~** 0.95; two-tailed *p*-value = 0.86).

To verify that the primary determinant of the observed diffusion effects was molecular size and not molecular identity, we compared the diffusion of dextran-MW70,000 (dextran-70K) to BSA (MW 67,000) (Fig. 4c). The diffusion dynamics of dextran-70K and BSA were similar; the two probes had diffusion τ_*D*_ values of 670 µs and 680 µs, respectively, in disorganized extracts, and the τ_*D*_ values for both decreased by a factor of almost 2 after self-organization (Fig. 4c). Collectively, these results suggest that self-organization specifically affects the short-range diffusion of protein-sized molecules.

### Proteins diffuse slightly slower in actin-intact extracts

So far, the experiments presented here have been carried out with extracts treated with the actin polymerization inhibitor cytochalasin B, a standard ingredient in extract preparation that helps keep the extracts pipettable^22,38^. However, it is possible to work with actin-intact extracts, and they are useful for interrogating processes that depend on actin or actomyosin^39^. Interphase actin-intact extracts self-organize, and the resulting morphology is similar to that seen in standard interphase extracts, although the border regions become somewhat wider by ~90 min^24^. Here we examined whether actin and actin-dependent motor activities might affect protein diffusion in organized cytoplasm, by comparing BSA diffusion in actin-intact extracts and in standard extracts.

**Fig. 5:**
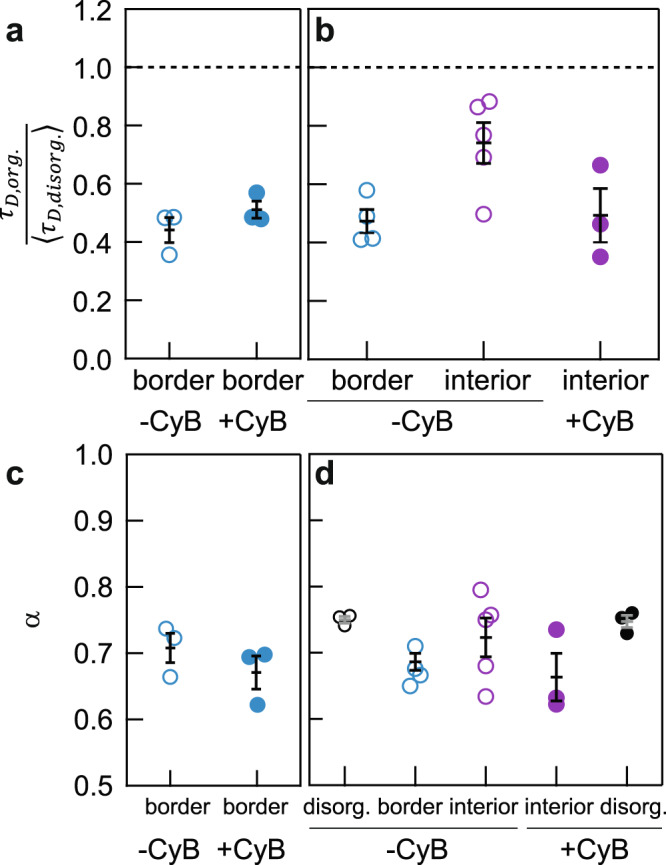
Protein diffusion in actin-intact extracts. **a**, **b** Normalized diffusion times of BSA in actin-intact extracts, prepared without the addition of cytochalasin B (CyB). Note that the diffusion rate did not increase more during the self-organization of actin-intact extracts (open circles) than it did in actin-inhibited extracts (solid circles). The two panels are two independent experiments. **c**, **d** Degree of subdiffusion in actin-intact and actin-inhibited extracts. Data shown in panel **c** and **d** are from the same experiment shown in panel **a** and **b**, respectively. Error bars, SEM. *n* = {3, 3, 4, 5, 6; 3, 3, 3, 4, 5, 6, 3} measurements of different positions in an extract for data from left to right and top to bottom, respectively. Source data are provided as a Source Data file.

In the border regions, we observed no difference in the degree of subdiffusion (*α*) and the diffusion time (τ_*D*_) of BSA between actin-intact and standard extracts (Fig. 5, Supplementary Fig. 9). In the interior regions, where actin was enriched^24^, we observed slightly slower diffusion in actin-intact extracts compared with actin-inhibited extracts, although the diffusion in the interior was still faster in the organized extracts than in the disorganized state (Fig. 5, Supplementary Fig. 9). In both actin-intact and standard extracts, BSA motions were subdiffusive with *α* **~** 0.6–0.8 (Fig. 5).

## Discussion

In summary, the present work shows that as interphase *Xenopus* egg extracts self-organize into cell-like units separated by organelle-depleted border regions, the diffusion of protein-sized probes (fluorescent BSA and dextran-70K) on an FCS distance scale (~hundreds of nm) becomes more rapid, approaching the dynamics seen in cytosolic extracts (Figs. 1, 2, 4). This occurs both in the border regions, where organelles and cytoskeletal elements are depleted during self-organization, and within the cell-like units, where these large obstacles are concentrated. The diffusion time (*τ*_*D*_) for the anomalous diffusion of protein-sized probes decreases by almost a factor of 2, and their effective diffusion coefficients increase by almost a factor of 2. This facilitation of protein diffusion is blocked by nocodazole, which inhibits microtubule polymerization and blocks self-organization, but not by the actin polymerization inhibitor cytochalasin B (Figs. 3, 5). No facilitation of diffusion is seen for a small molecule probe (Alexa Fluor 488) or for a 2 MDa labeled dextran (Fig. 4). On a particle-tracking distance scale (microns), 100 nm microspheres show no facilitation of their diffusion after cytoplasmic organization; in fact, they diffuse more slowly (Fig. 1d). Taken together, these findings suggest that cytoplasmic self-organization maximizes the speed at which protein-sized molecules traverse distances of relevance to signal transduction and dynamical protein-protein interactions.

Several mechanisms can be envisioned to account for the observed facilitation of diffusion. First, it is possible that self-organization makes the stirring of the cytoplasm by motor proteins or other ATPases more efficient. Several previous studies have shown that ATP is critical for the diffusion of various large particles in cells, including microspheres of hundreds of nanometers^40^, genetically encoded multimeric nanoparticles (GEM) of 20- and 40-nm^41^, chromosomal loci^42^, and filaments, plasmids, and granules^4^, and that the activity of myosin II motors on actin filaments contributes to this facilitation^40^. It is possible that something similar could contribute to the increase in protein diffusion that accompanies cytoplasmic self-organization, although it would probably not involve actomyosin, since inhibiting actin polymerization did not lessen the facilitation of diffusion seen during self-organization (and in fact slightly increased it; Fig. 5).

We have not been able to directly test whether ATP depletion affects the dynamics of proteins in organized cytoplasm, since the self-organization itself requires ATP^24^. However, several observations argue against cytoplasmic stirring being relevant to the changes in protein diffusion that accompany self-organization. First, the probes whose dynamics are known to depend upon cytoplasmic stirring are generally substantially larger than BSA and dextran-70 K. For example, the study that demonstrated ATP-dependent motions for 100-nm microspheres (measured by SPT) found that ATP-depletion had no measurable effect on a 27 kDa protein (GFP) diffusing over a shorter distance (measured by FCS)^40^. That study also implicated actomyosin activity as being important for cytoplasmic stirring, whereas here we found that blocking actin polymerization slightly increased, rather than decreased, protein diffusion after self-organization. It is possible that the contraction of the cell-like units that take place when actin filaments are present increases the concentration of obstacles^24,43^, accounting for the slower protein diffusion. The slower diffusion observed in actin-intact extracts is consistent with mobility studies of quantum dots (14-nm radius) and GFP trimers in actin-intact versus actin-inhibited cells^44,45^ but not with studies of larger particles (40-nm GEM) in actin-perturbed cells^46^. Moreover, if stirring is due to microtubule motors like dynein and kinesin, or general ATPases, stirring might be maximal well before self-organization, and the accompanying facilitation of protein diffusion, is completed. General ATPases would be expected to be active as soon as an extract is warmed to room temperature, and microtubule asters are typically maximal in size within 5–10 min of warming^24^. If this is true, then ATPase-dependent stirring might contribute about equally to the dynamics of both disorganized and organized extracts. Finally, protein motions we observed were all subdiffusive (*α* < 0.85), in contrast to the characteristic Brownian-like motions for active diffusion found in previous reports^40^. Thus, it seems likely that mechanisms additional to active diffusion must facilitate the change in protein diffusion that accompanies self-organization.

**Fig. 6:**
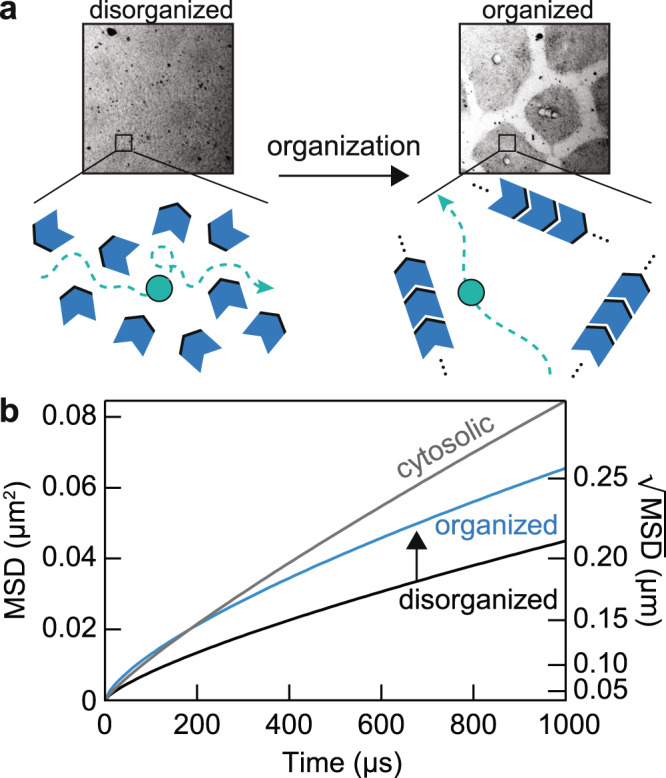
Organized cytoplasm optimizes short-range diffusion by reorganizing obstacle densities. **a** Schematic of the model: molecular and organelle reorganization optimizes molecular diffusion by creating longer mean free paths for diffusing molecules. The green object represents a protein of interest diffusing in the cytoplasm; the blue objects represent obstacles in the cytoplasm, such as organelles, cytoskeleton elements, and macromolecular complexes. In this model, proteins in organized cytoplasm remain free to diffuse in all directions. **b** Diffusion effects of molecular reorganization illustrated with mean squared displacement, *MSD* (*t*) = Γ*t*^*α*^. The parameters {Γ, *α*} estimated for disorganized cytoplasm, organized cytoplasm and the cytosol are {8, 0.75}, {8.25, 0.70}, and {30, 0.85}, respectively. Note that based on the tradeoff between diffusion times and subdiffusivity in the organizing cytoplasm (Fig. 3a), we have assumed that the acceleration of protein movements over these distance scales comes at a cost of slower dynamics over longer distance scales. We also limit the comparison around the length scale of FCS measurements (~0.1–0.5 µm) since protein dynamics may show distinct characteristics at shorter and longer distance scales. Source data are provided as a Source Data file.

We propose that a more efficient packing of organelles and molecules in the organized cytoplasm facilitates the diffusion of protein molecules in the cytosol. The coalescence of organelles and molecules in the organized cytoplasm reduces the obstacle density locally, so that protein-sized molecules can more freely diffuse over distances of a few hundred nanometers. This hypothesis is shown schematically in Fig. 6a. Although diffusing proteins encounter fewer collisions locally, we envision that the organized structures hinder long-range diffusion more than short-range diffusion, accounting for the trade-off between *τ*_*D*_ and *α* seen in Fig. 3a. The net result can be illustrated with a mean-squared displacement plot (Fig. 6b). Over a time span of ~500 µs and a distance scale of ~200 nm, the mean squared displacement for a protein in organized cytoplasm is at least 85% of that measured for cytosol. As previously mentioned, a protein *X* would need to travel about 200 nm on average to find a moderately-scarce protein *Y*. Thus, the time it takes for *X* to find *Y*, or any less-scarce target, would be almost as fast in organized cytoplasm as it would be in organelle-depleted cytosol.

Conceptually, this description of proteins diffusing in the organized cytoplasm is reminiscent of the Lorentz model, which is one of several ways of accounting for anomalous diffusion^34^. The Lorentz model considers the cytoplasm to be a heterogeneous dispersion of biomolecules, rather than a homogeneous phase as assumed in several other anomalous diffusion models. This feature of the Lorentz model is especially relevant in the context of cytoplasmic organization, in which an increase in the degree of organization maps to a decrease in the obstacle density. Thus, a diffusing probe encounters fewer obstacles in the organized cytoplasm and is able to diffuse faster over a short distance. Similar descriptions based on the Lorentz model have been developed from multi-scale FCS in living cells: the diffusing pattern of GFP in the cytoplasm resembles percolation through a random obstacle structure that maps to a porous medium topology^44^.

More broadly, the anomalous diffusion (for protein-sized molecules, *α* ≈ 0.6–0.8) and effective diffusion coefficients (*D*_*eff*_ ≈ 10–20 µm^2^/s) in this study generally agree well with live-cell measurements^32,33,35^, suggesting that egg extracts recapitulate the physical properties of living cytoplasm. The present work is also generally consistent with previous studies of the biophysical properties of extracts^47,48^, although in retrospect it is not clear how organized the extracts used in those studies were.

Although the cytoplasm in living cells undergoes dramatic reorganization during the cell cycle, the disorganized extracts examined here are likely to be even more well-mixed and homogenized than post-mitotic extracts or cells. This is suggested by the observation that after one or a few cycles, cycling extracts generally reorganize faster after M-phase exit than interphase-arrested extracts do^24,29^. Likewise, homogenized interphase extracts may be more disorganized than somatic cells exiting mitosis, or nocodazole-treated somatic cells, which are constrained by the toxicity of high doses of nocodazole^49^. This may explain why changes in diffusivity were not obvious after microtubule inhibitor treatment in some cell-based studies^33,44,50^. It is also possible that microtubules are required to establish but not to maintain cytoplasmic structures that act as diffusion barriers in cells, such as the ER network^51,52^. If so, cytoskeleton inhibitors may not fully abolish the pre-established organization that is inherent in living cells prior to drug treatment. Thus, the extract’s ability to self-organize from a highly disorganized state makes extracts an advantageous model system to elucidate the kinetic effects of cytoplasmic organization.

In cells, biochemical reactions must proceed efficiently enough despite the constrained diffusivity. Several mechanisms that facilitate molecular motions have been proposed, such as motor-based transport and active diffusion^40,53,54^, macromolecule tuning^14,46,55^, biomolecular condensation^46,56,57^, and intracellular pH changes^58^. These mechanisms, and ours, are not mutually exclusive, and may simultaneously operate on different length scales and timescales. Nevertheless, for protein-sized molecules and biochemically relevant time scales, cytoplasmic organization constitutes an important factor in determining biomolecular dynamics.

## Methods

### *Xenopus laevis* egg extracts

All *Xenopus* experiments and animal care followed protocols (APLAC-13307) approved by the Institutional Animal Care and Use Committee (IACUC) of Stanford University. *Xenopus laevis* frogs were 3 years old from Nasco. Interphase-arrested cytoplasmic extracts and cytosolic extracts were prepared following the protocol of Deming and Kornbluth, except without the energy regeneration mix^24,27,28^. Cytoplasmic extracts were placed on ice before imaging on the same day; cytosolic extracts were flash-frozen and kept in a −80 °C freezer before use. Demembranated sperm nuclei were prepared as described^27^, and added into the cytoplasmic extracts at a final concentration of about 160 nuclei/µL. ER-Tracker Red dye (ThermoFisher) was added at a final concentration of 1 µM to visualize the endoplasmic reticulum (ER). The extracts were further supplemented with cOmplete EDTA-free protease inhibitor cocktail (Roche) at the recommended concentration (1x) and incubated for 30 min on ice prior to imaging. For experiments that inhibited self-organization, we added nocodazole (MilliporeSigma) at a final concentration of 33 µM.

One of the desired diffusion probes was added to an extract right before imaging at the indicated final concentration: 25 nM albumin from bovine serum with Alexa Fluor 488 conjugate (BSA-AF488) (Invitrogen), 25 nM Alexa Fluor 488 (Invitrogen), 25 nM dextran-fluorescein-MW70,000 (dextran-70 K) (Invitrogen), 10 nM dextran-fluorescein-MW2,000,000 (dextran-2M) (Invitrogen), or 0.1-µm TetraSpeck microspheres (Invitrogen). All probes, except the microspheres, were centrifuged at 16,000 g for 5 min to reduce aggregates before adding to extracts. Extracts were mixed thoroughly during each step. Extracts (2 µL) were then dropped on a bioinert µ-Dish 35 mm imaging chamber (No. 1.5) (ibidi) and immediately covered with heavy mineral oil (MilliporeSigma). For experiments that compared different conditions, each imaging dish contained 2–3 drops of well-separated extracts. The extracts formed similar self-organized structures in this simple setup, compared to the previous chamber setup that sandwiched the extracts with a defined spacing^28^. We typically began the imaging session within 3 min after extracts were taken off the ice, and usually within 1 h after crushing the eggs.

### Confocal and FCS microscopy

All confocal images and FCS data were collected with an inverted Zeiss LSM 780 multiphoton laser scanning confocal microscope at room temperature (22 °C). Samples were excited by lasers that passed through dichroic mirrors and focused by a C-APO 40x (FCS-certified) water-based objective. Signals were collected by the LSM BiG module with GaAsP photodetector, which enabled the selection of detection wavelength. For extract experiments, we typically focused the confocal spot about 30–40 µm above the dish surface, which often placed the nuclei near the focal plane. All data were acquired using the ZEN 2.3 SP1 FP3 (Black) software (version 14) (Zeiss).

For FCS, samples were excited by a 488 nm laser line at 5 µW that passed through a 488 beam splitter. Emissions passed through a variable secondary dichroic (VSD) beam splitter that selected signals from wavelength 499–579 nm. The pinhole was aligned using the “Adjust pinhole” function in the software. The spot-size was calibrated by averaging spot-sizes determined by fluorescein (Sigma Aldrich) (*D* = 425 µm^2^/s)^59^, Alexa Fluor 488 (Invitrogen) (*D* = 435 µm^2^/s)^60^, Atto488-carboxylic acid (Atto-Tec) (*D* = 400 µm^2^/s) (PicoQuant application note by Peter Kapusta, https://www.picoquant.com/images/uploads/page/files/7353/appnote_diffusioncoefficients.pdf), and 0.1 µm TetraSpeck microspheres (Invitrogen) (*D* = 4.4 µm^2^/s)^61^ in water at room temperature assuming known diffusion coefficients. For calibration, each FCS curve was acquired for 5 min. For extracts, each data point corresponds to an averaged FCS curve taken from 60 s of data acquisition; in rare instances (<5%), irregular FCS curves, such as those caused by aggregates, were removed from the analysis. All averaging was calculated in the ZEN software. Most data focused on one self-organizing unit with morphology similar to that shown in Fig. 2a and its immediate surrounding units. All data points were different positions taken within the same region (as defined in Fig. 2a); the only exception that reports measurements from the same position was the quantification of the FCS measurement error (Supplementary Fig. 5).

For confocal images, samples were excited by a 561 nm laser line at 72 µW that passed through an MBS488/561 dichroic mirror. Emissions were collected by integrating signals from wavelength 578–695 nm. The pixel time was 2.55 µs and the gain was set to 700. Each confocal image was collected by scanning 512 × 512 pixels, corresponding to 354 × 354 µm. Tile-scan combined 5 × 5 adjacent images, resulting in 2354 × 2354 pixels (1630 × 1630 µm).

### FCS analysis

The ZEN program calculated time autocorrelation functions by ${G}_{{ZEN}}(\tau )=\frac{\left\langle I\left(t\right)I\left(t+\tau \right)\right\rangle }{{\left\langle I\left(t\right)\right\rangle }^{2}}$, where *I*(*t*) is the fluorescence intensity, *τ* is the delay time, and $\left\langle \bullet \right\rangle$ denotes the average. This function relates to our definition of autocorrelation function by $G(\tau )=\frac{\left\langle \delta I\left(t\right)\delta I\left(t+\tau \right)\right\rangle }{{\left\langle I\left(t\right)\right\rangle }^{2}}={G}_{{ZEN}}(\tau )-1$, where $\delta I\left(t\right)=I\left(t\right)-\left\langle I\left(t\right)\right\rangle$^31^. All subsequent fittings were performed in Igor Pro (version 6). FCS data were fitted with a 3D anomalous diffusion model^34^:

$$G(\tau )=\frac{1}{N\left(1+{\left(\frac{\tau }{{\tau }_{D}}\right)}^{\alpha }\right)\sqrt{\left(1+\frac{1}{{s}^{2}}{\left(\frac{\tau }{{\tau }_{D}}\right)}^{\alpha }\right)}}$$

where *α* is the diffusion-mode parameter (as defined in the MSD equation, ${MSD}(t)\propto {t}^{\alpha }$), *τ*_*D*_ is the characteristic diffusion time, *N* is the particle number, and *s* is the structural parameter of the optics. For calibrations, the data were fitted to a Brownian model, with *α* *=* 1. For most FCS curves, we fitted a time range of 10 µs-0.1 s; for faster (free dyes) or slower (dextran-2M) diffusion, we expanded the fitting range down to 1 µs or up to 3 s, respectively. In our experimental conditions, we did not see notable triplet-state dynamics for the probes we have used (little to no correlation decay in the ~1–10 µs time lag; and our fitting range started from 10 µs). To calculate an effective diffusion coefficient from anomalous diffusion fits, we used the relationship, ${D}_{{eff}}={w}^{2}/4{\tau }_{D}$ where *w* is the confocal spot radius and *τ*_*D*_ is the measured diffusion time.

### Single-particle tracking

Tracking experiments were performed on a Nikon TiE inverted microscope. Samples containing 100-nm (diameter) TetraSpeck microspheres (Invitrogen) were excited by a 475/28-nm light source (Lumencor Spectra III). The excitation light passed through a 474/27-nm bandpass filter and a 493-nm dichroic mirror (Sedat quad filter set, Semrock) and focused through a 60 × 1.40 N.A. oil immersion objective. The emission signals passed through a 528/38-nm bandpass filter (Sedat quad filter set, Semrock) and were collected by an Andor EM-CCD camera (iXon DU-897). Images were acquired at 25 fps for 60 s with an exposure time of 10 ms. All data acquisitions were controlled by the NIS-Elements software (version 5.11.03, build 1373).

Tracking analysis was done using TrackMate^62^ in ImageJ using difference of gaussian (DoG) detection algorithm. The detected particles were then linked by the simple linear assignment problem (LAP) trackers algorithm, which did not allow any branched nor merged tracks. The maximum travel distance was limited to 2 µm with a maximum frame interval between two spots limited to 2. The maximum travel distance was cross-verified with the diffusion coefficient obtained from step-size distribution analysis. Tracks >1 s were then exported for further analysis. Ensemble MSD was obtained from trajectories using the following equation in Matlab (R2017a): ${MSD}(j\triangle t)=\frac{1}{N}{\sum }_{i=1}^{N}[{({x}_{i}(j\triangle t)-{x}_{i}(0))}^{2}+{({y}_{i}(j\triangle t)-{y}_{i}(0))}^{2}]$ where $\{{x}_{i}\left(t\right),{y}_{i}\left(t\right)\}$ is the position of trajectory *i*, *j* is the frame number, Δ*t* is the time interval between frames, and *N* is the total number of trajectories. Figure 1d was analyzed from 505 trajectories and 101 trajectories of tracked duration >1 s in disorganized and organized extracts, respectively (another movie of 126 trajectories in the same organized extract confirms the slower diffusion in organized extracts); Supplementary Fig. 1a was analyzed from 239 trajectories of tracked duration >1 s.

### Statistics and reproducibility

The primary experiment measuring BSA diffusion in cytoplasmic extracts has been repeated more than six times. All other experiments were repeated 2–3 times. If the egg quality was good (i.e., nonapoptotic), all the repeats yielded consistent results. We used Mann–Whitney *U*-tests (also known as the Wilcoxon rank-sum test) for all data comparison since the Mann–Whitney test is a nonparametric method that makes no assumption of the distribution. The results of the statistical tests are presented in Supplementary Fig. 6, 8 and 9. All comparisons were analyzed from experiments on the same day using the same batch of freshly prepared egg extracts. We have also compared the data using Welch’s *t*-test (unequal variance) and the Kolmogorov–Smirnov test and reached the same statistical conclusions.

### Reporting summary

Further information on research design is available in the Nature Research Reporting Summary linked to this article.

## Supplementary information

Supplementary Information

Reporting Summary

## Data Availability

Data generated in this study have been deposited in the Stanford Digital Repository at https://purl.stanford.edu/fb711ym3445. Source data are provided with this paper.

## Source data

Source Data
