# Supplementary material for: Cytoplasmic organization promotes protein diffusion in Xenopus extracts
Source: Nat Commun. 2022 Sep 23;13:5599. doi: 10.1038/s41467-022-33339-0 (PMC9508076; doi:10.1038/s41467-022-33339-0)
Supplement: Supplementary file 1 — Supplementary Information [file 41467_2022_33339_MOESM1_ESM.pdf]

Supplementary Information for
Cytoplasmic organization promotes protein diffusion in *Xenopus* extracts

William Y. C. Huang *et al.*

Supplementary Figures

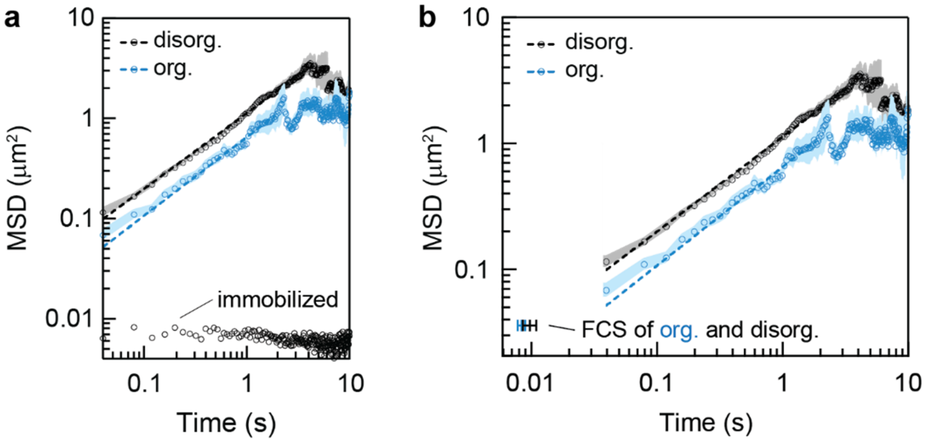

Supplementary Figure 1: Localization error in SPT and comparison with FCS.
a, Data from Fig. 1d overlaid with MSD of immobilized microspheres on glass. This shows that the localization error was below 0.01 µm^2, which was lower than the mobile particles in extracts. **b,** Data from Fig. 1d overlaid with the dextran-2M FCS data from Fig. 4. The SPT data was analyzed for 100-nm microspheres and the FCS data was obtained for dextran-2M (diameter of ~90 nm). Error bars, SEM (standard error of the mean).

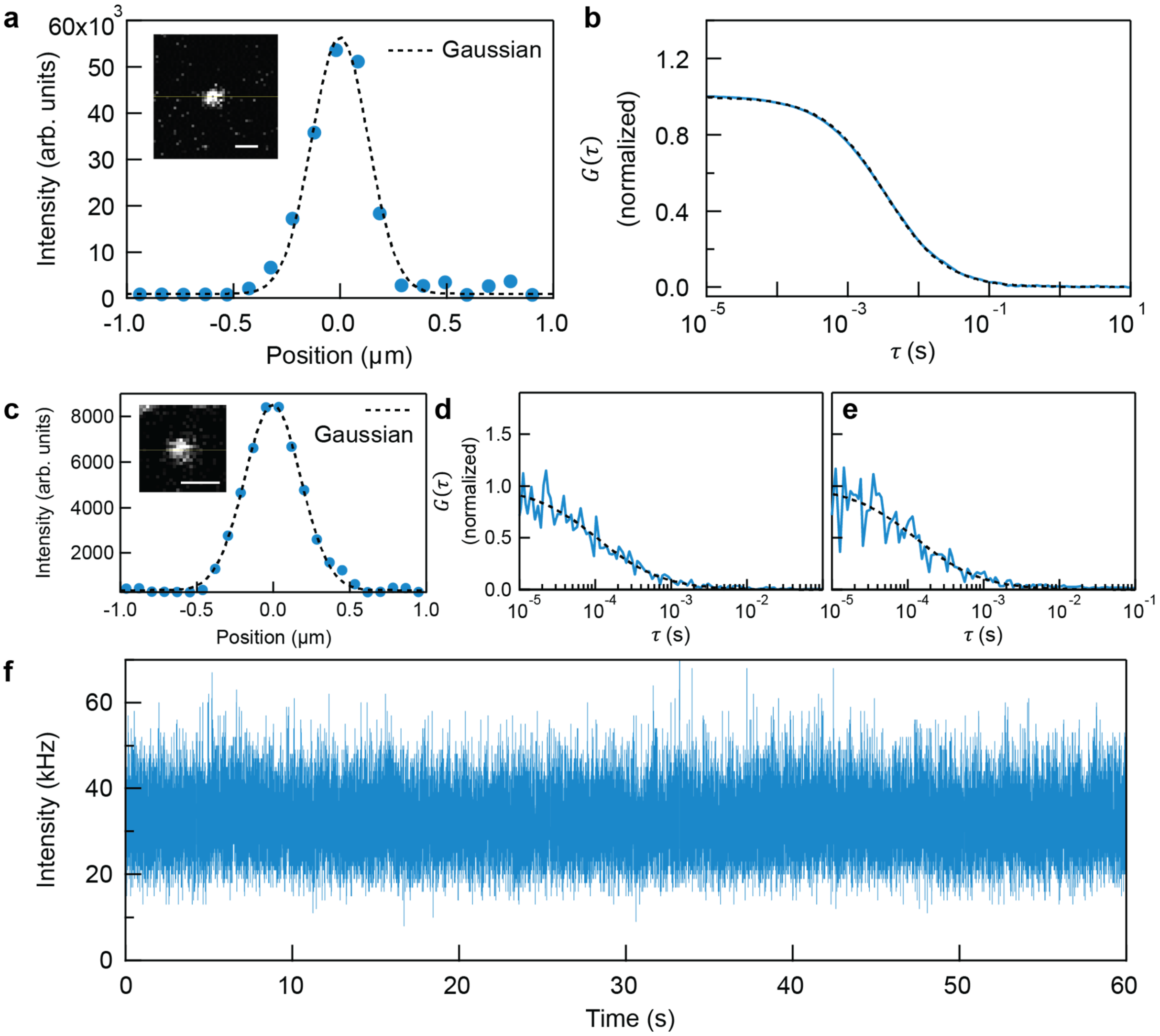

Supplementary Figure 2: FCS calibration.

a, Estimated point-spread function (PSF) imaged by scanning a 100-nm immobilized microsphere on a glass substrate. These microspheres were chosen because of their exceptionally high molecular brightness (~300-fold brighter than BSA-AF488), which makes them ideal for characterizing the PSF and α. Scale bar, 1 µm. **b,** FCS curve of the identical microsphere but in water. Dashed line, fitting to Brownian diffusion. Fitting to an anomalous diffusion yielded $\alpha = 0.97$, suggesting that imperfection in optics did not notably distort the type of diffusion. **c,** Same as **a** but measured in a disorganized extract. **d, e,** FCS curves of Alexa Fluor 488 in disorganized and organized extracts, respectively. Dashed lines, fitting to Brownian diffusion. Fitting to an anomalous diffusion yielded $\alpha = 0.98 \pm 0.06$ (±SEM, 3 data points) and 0.94 ± 0.03 (4 data points), respectively. **f,** Full intensity trajectory of data shown in Fig. 1e.

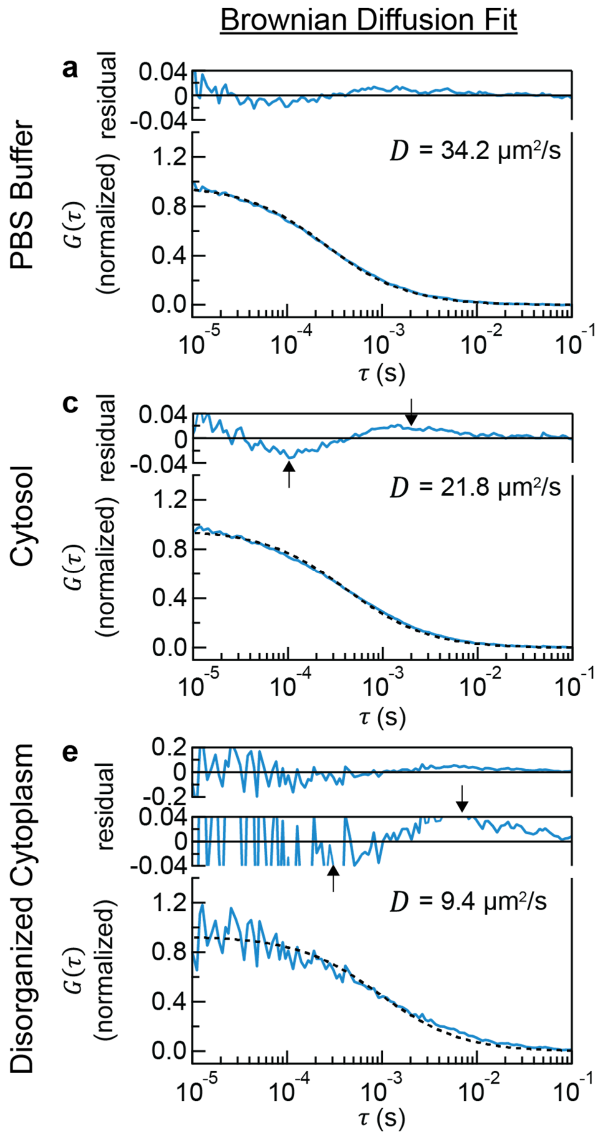

Supplementary Figure 3: Diffusion analysis of FCS autocorrelation functions.
a, **c**, **e**, Fitting a Brownian model to data for BSA diffusion in PBS buffers, cytosolic extracts, and disordered cytoplasmic extracts. **b**, **d**, **f**, Fitting an anomalous diffusion model to the same data. Note that Brownian motion ideally yields $\alpha = 1$ even with anomalous diffusion fits (also see Supplementary Fig. 2b).

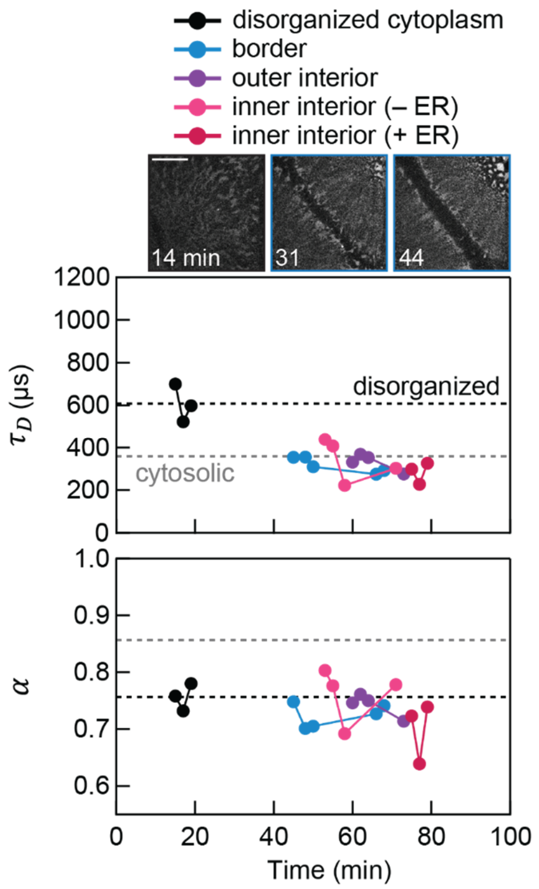

Supplementary Figure 4: Dynamical transition in cytoplasmic extracts: an example of extracts self-organized slightly earlier.

A biological repeat of Fig. 2 experiments. In this sample, the cytoplasm formed organized structures earlier than the other experiments; as a result, the diffusion changes between the disorganized and organized states were especially apparent. Note that this extract formed cell-like pattern earlier than other examples, leading to earlier transition to faster diffusion.

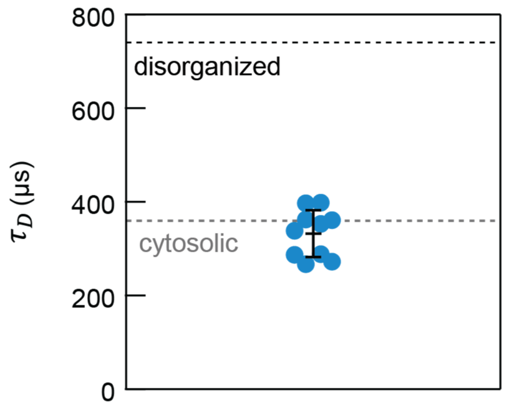

Supplementary Figure 5: Measurement error in cytoplasmic extracts.
Estimation of the upper bound of measurement error in cytoplasmic extracts. The data show n = 10 repetitive measurements at the same position in a mature border; each measurement was acquired identically as Fig. 2c and e. The error bar shows the standard deviation (50 µs) around the mean. Note that since cytoplasmic extracts are highly dynamic and non-stationary (unlike homogeneous samples), this error includes measurement error and fluctuations of cytoplasmic organization. Source data are provided as a Source Data file.

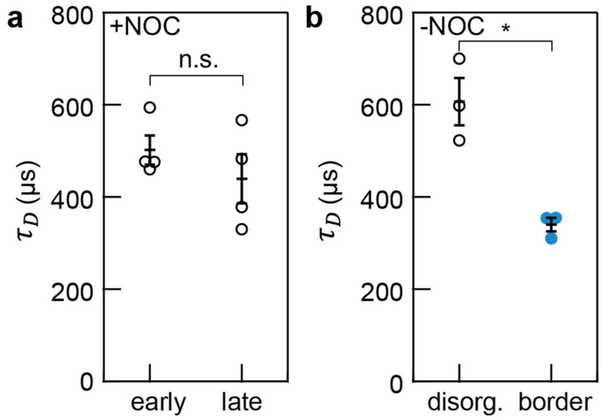

Supplementary Figure 6: Statistical tests of BSA diffusion in microtubule-perturbed cytoplasmic extracts.

Mann–Whitney U tests for data in Fig. 3c. Each panel are data measured on the same day. Asterisks (*) denotes the significance levels calculated as two-tailed p-values: *, $p \leq 0.15$; n.s., not significant, $p > 0.15$. The p-values for **a** and **b** are 0.686, 0.100, respectively. Error bars, SEM. *n* = 4, 4, 3, and 3 measurements of different positions in an extract for data from left to right, respectively. Source data are provided as a Source Data file.

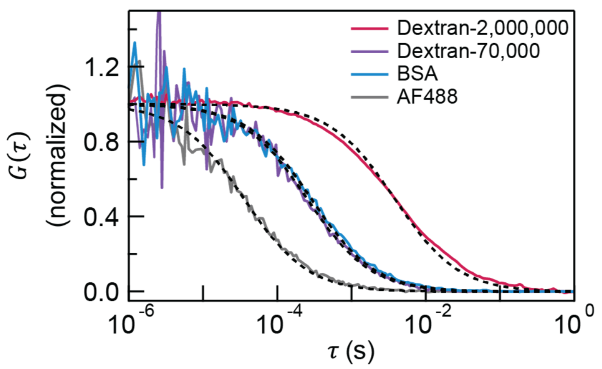

Supplementary Figure 7: Size estimation of probes used in this study.

The relative sizes of the diffusion probes were measured in PBS buffers using FCS. The autocorrelation functions were fitted by a Brownian model ($\boldsymbol{\alpha = 1}$) (dashed curves). The diffusion times yield the relative Stokes radii by the Stokes-Einstein equation, $\boldsymbol{D \propto 1/R}$. The relative Stokes radii for AF488, BSA, dextran-70K, and dextran-2M are 0.12, 1.00, 0.88, and 13.3, respectively. The relative values were converted to absolute lengths by taking the Stokes radius of BSA to be 3.5 nm. As a note, the polydisperse nature of dextran-2M could also be effectively fitted by the anomalous diffusion model, though the diffusion was expected to be Brownian[11]. Nevertheless, the fitted diffusion times between the two models were similar.

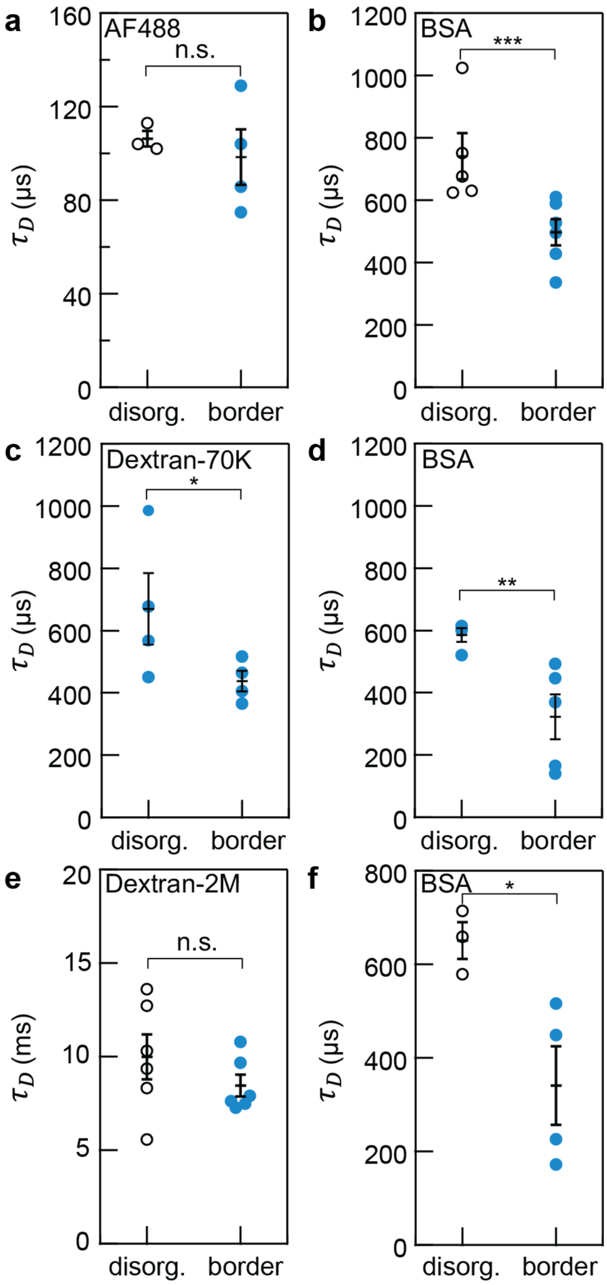

Supplementary Figure 8: Statistical tests of particle diffusion in cytoplasmic extracts. Mann–Whitney U tests for data in Fig. 4. Each panel are data measured on the same day. Asterisks (*) denotes the significance levels calculated as two-tailed p-values: ***, $p \leq 0.01$; **, $p \leq 0.05$; *, $p \leq 0.15$; n.s., not significant, $p > 0.15$. The p-values for **a-f** are 0.686, 0.004, 0.114, 0.016, 0.310, and 0.057, respectively. Error bars, SEM. n = {3, 4, 5, 6; 4, 4, 3, 5; 6, 6, 3, 4} measurements of different positions in an extract for data from left to right and top to bottom, respectively. Source data are provided as a Source Data file.

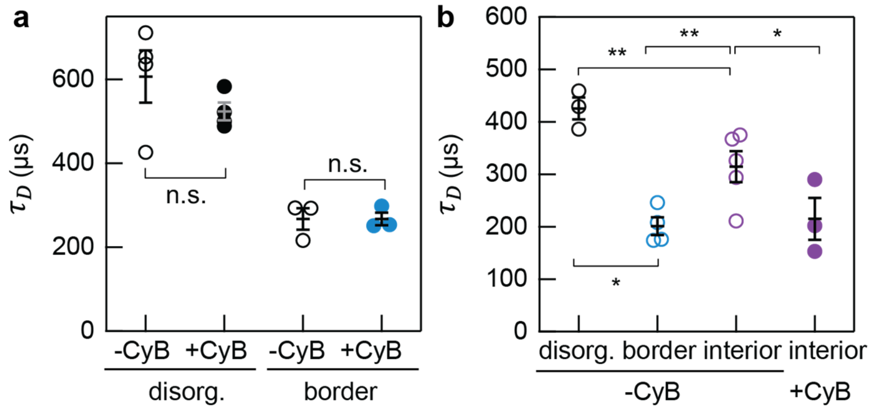

Supplementary Figure 9: Statistical tests of BSA diffusion in actin-intact cytoplasmic extracts.

Mann–Whitney U tests for data in Fig. 5. Asterisks (*) denotes the significance levels calculated as two-tailed p-values: **, $p \leq 0.05$; *, $p \leq 0.15$; n.s., not significant, $p > 0.15$. The p-values for **a** and **b** are {0.34, 1.00} (from left to right), and {0.036, 0.032, 0.071, 0.057} (from top left to top right to bottom), respectively. The two panels show data from two independent experiments. Error bars, SEM. n = 4, 3, 3, 3, 3, 4, 5, and 3 measurements of different positions in an extract for data from left to right, respectively. Source data are provided as a Source Data file.

Supplementary References

1 Cheng, X. & Ferrell, J. E. *Xenopus* laevis egg extract preparation and live imaging methods for visualizing dynamic cytoplasmic organization. *Journal of visualized experiments : JoVE*, doi:10.3791/61923 (2021).

2 Deming, P. & Kornbluth, S. Study of apoptosis in vitro using the *Xenopus* egg extract reconstitution system. *Methods in Molecular Biology* **322**, 379-393 (2006).

3 Cheng, X. & Ferrell, J. E. Spontaneous emergence of cell-like organization in *Xenopus* egg extracts. *Science* **366**, 631-637, doi:10.1126/science.aav7793 (2019).

4 Culbertson, C. T., Jacobson, S. C. & Michael Ramsey, J. Diffusion coefficient measurements in microfluidic devices. *Talanta* **56**, 365-373, doi:10.1016/s0039-9140(01)00602-6 (2002).

5 Petrasek, Z. & Schwille, P. Precise measurement of diffusion coefficients using scanning fluorescence correlation spectroscopy. *Biophys. J.* **94**, 1437-1448, doi:DOI 10.1529/biophysj.107.108811 (2008).

6 Dertinger T., E. B. *PicoQuant GmbH* (2008).

7 Muller, C. B., Weiss, K., Richtering, W., Loman, A. & Enderlein, J. Calibrating differential interference contrast microscopy with dual-focus fluorescence correlation spectroscopy. *Opt Express* **16**, 4322-4329, doi:10.1364/oe.16.004322 (2008).

8 Krichevsky, O. & Bonnet, G. Fluorescence correlation spectroscopy: the technique and its applications. *Rep. Prog. Phys.* **65**, 251-297, doi:10.1088/0034-4885/65/2/203 (2002).

9 Hofling, F. & Franosch, T. Anomalous transport in the crowded world of biological cells. *Reports on progress in physics. Physical Society* **76**, 046602, doi:10.1088/0034-4885/76/4/046602 (2013).

10 Tinevez, J. Y. *et al.* TrackMate: An open and extensible platform for single-particle tracking. *Methods* **115**, 80-90, doi:10.1016/j.ymeth.2016.09.016 (2017).

11 Kalwarczyk, T. *et al.* Apparent anomalous diffusion in the cytoplasm of human cells: the effect of probes' polydispersity. *J. Phys. Chem. B* **121**, 9831-9837, doi:10.1021/acs.jpcb.7b07158 (2017).
