# Supplementary material for: Cytoplasmic organization promotes protein diffusion in Xenopus extracts
Source: Nat Commun. 2022 Sep 23;13:5599. doi: 10.1038/s41467-022-33339-0 (PMC9508076; doi:10.1038/s41467-022-33339-0)
Supplement: Supplementary file 2 — Reporting Summary [file 41467_2022_33339_MOESM2_ESM.pdf]

Corresponding author(s): Xianrui Cheng
James E. Ferrell

Last updated by author(s): 8/25/2022

Reporting Summary

Nature Portfolio wishes to improve the reproducibility of the work that we publish. This form provides structure for consistency and transparency in reporting. For further information on Nature Portfolio policies, see our Editorial Policies and the Editorial Policy Checklist.

Statistics

For all statistical analyses, confirm that the following items are present in the figure legend, table legend, main text, or Methods section.

n/a	Confirmed	
☐	☒	The exact sample size (*n*) for each experimental group/condition, given as a discrete number and unit of measurement
☐	☒	A statement on whether measurements were taken from distinct samples or whether the same sample was measured repeatedly
☐	☒	The statistical test(s) used AND whether they are one- or two-sided<br>*Only common tests should be described solely by name; describe more complex techniques in the Methods section.*
☒	☐	A description of all covariates tested
☒	☐	A description of any assumptions or corrections, such as tests of normality and adjustment for multiple comparisons
☐	☒	A full description of the statistical parameters including central tendency (e.g. means) or other basic estimates (e.g. regression coefficient) AND variation (e.g. standard deviation) or associated estimates of uncertainty (e.g. confidence intervals)
☐	☒	For null hypothesis testing, the test statistic (e.g. *F*, *t*, *r*) with confidence intervals, effect sizes, degrees of freedom and *P* value noted<br>*Give P values as exact values whenever suitable.*
☒	☐	For Bayesian analysis, information on the choice of priors and Markov chain Monte Carlo settings
☒	☐	For hierarchical and complex designs, identification of the appropriate level for tests and full reporting of outcomes
☒	☐	Estimates of effect sizes (e.g. Cohen's *d*, Pearson's *r*), indicating how they were calculated

Our web collection on statistics for biologists contains articles on many of the points above.

Software and code

Policy information about availability of computer code

Data collection	All fluorescence correlation spectroscopy (FCS) data were collected with an inverted Zeiss LSM 780 multiphoton laser scanning microscope controlled by the ZEN 2.3 SP1 FP3 (black) software (version 14) (Zeiss). All single-particle tracking data were collected with an Nikon TiE inverted microscope controlled by the NIS-Elements software (version 5.11.03, build 1373).
Data analysis	All fittings and statistical tests were performed in Igor Pro (version 6).

For manuscripts utilizing custom algorithms or software that are central to the research but not yet described in published literature, software must be made available to editors and reviewers. We strongly encourage code deposition in a community repository (e.g. GitHub). See the Nature Portfolio guidelines for submitting code & software for further information.

Data

Policy information about availability of data

All manuscripts must include a data availability statement. This statement should provide the following information, where applicable:

- Accession codes, unique identifiers, or web links for publicly available datasets
- A description of any restrictions on data availability
- For clinical datasets or third party data, please ensure that the statement adheres to our policy

All data needed to evaluate the conclusions in the paper are present in the paper or the supplementary information. Data generated in this study are available in the Stanford Digital Repository (https://purl.stanford.edu/fb711ym3445).

Human research participants

Policy information about studies involving human research participants and Sex and Gender in Research.

Reporting on sex and gender	No human research participants involved in this study.
Population characteristics	n/a
Recruitment	n/a
Ethics oversight	n/a

Note that full information on the approval of the study protocol must also be provided in the manuscript.

Field-specific reporting

Please select the one below that is the best fit for your research. If you are not sure, read the appropriate sections before making your selection.

[x] Life sciences   [ ] Behavioural & social sciences   [ ] Ecological, evolutionary & environmental sciences

For a reference copy of the document with all sections, see nature.com/documents/nr-reporting-summary-flat.pdf

Life sciences study design

All studies must disclose on these points even when the disclosure is negative.

Sample size	Sample size was chosen by maximizing the number of independent positions while the extract was in a similar state (<10 min); this number was also limited by a minimum of 1 min acquisition time necessary to obtain reliable FCS data.
Data exclusions	No data were excluded from the analyses.
Replication	All experiments were repeated at least twice; the primary experiment (Fig.2) was repeated more than six times. All repeats yield the same conclusions.
Randomization	This is not relevant to this study. This study did not involve grouping of samples, organisms, or participants.
Blinding	Blinding was not relevant to this study because no experimental groups were involved.

Reporting for specific materials, systems and methods

We require information from authors about some types of materials, experimental systems and methods used in many studies. Here, indicate whether each material, system or method listed is relevant to your study. If you are not sure if a list item applies to your research, read the appropriate section before selecting a response.

Materials & experimental systems

n/a	Involved in the study	
[x]	[ ]	Antibodies
[x]	[ ]	Eukaryotic cell lines
[x]	[ ]	Palaeontology and archaeology
[ ]	[x]	Animals and other organisms
[x]	[ ]	Clinical data
[x]	[ ]	Dual use research of concern

Methods

n/a	Involved in the study	
[x]	[ ]	ChIP-seq
[x]	[ ]	Flow cytometry
[x]	[ ]	MRI-based neuroimaging

Animals and other research organisms

Policy information about studies involving animals; ARRIVE guidelines recommended for reporting animal research, and Sex and Gender in Research

Laboratory animals	Xenopus laevis frogs (3 years old; lab-bred strain from Nasco)
Wild animals	This study did not involve wild animals

Reporting on sex	Male and female
Field-collected samples	This study did not involve field-collected samples
Ethics oversight	All Xenopus experiments and animal care followed protocols (APLAC-13307) approved by the Institutional Animal Care and Use Committee (IACUC) of Stanford University.

Note that full information on the approval of the study protocol must also be provided in the manuscript.
